# Impact of hospital nephrectomy volume on intermediate‐ to long‐term survival in renal cell carcinoma

**DOI:** 10.1111/bju.14848

**Published:** 2019-07-15

**Authors:** Ray C. J. Hsu, Matthew Barclay, Molly A. Loughran, Georgios Lyratzopoulos, Vincent J. Gnanapragasam, James N. Armitage

**Affiliations:** ^1^ Academic Urology Group Department of Surgery Cambridge Biomedical Campus University of Cambridge Cambridge UK; ^2^ Department of Urology Addenbrooke's Hospital Cambridge University Hospitals NHS Foundation Trust Cambridge UK; ^3^ The Healthcare Improvement Studies (THIS) Institute University of Cambridge Cambridge UK; ^4^ Transforming Cancer Services Team National Health Service London UK; ^5^ National Cancer Registration and Analysis Service Public Health England London UK; ^6^ Epidemiology of Cancer Healthcare and Outcomes (ECHO) Group Department of Behavioural Science and Health University College London London UK

**Keywords:** renal cell carcinoma, nephrectomy, centralisation, survival, hospital volume, #KidneyCancer, #kcsm

## Abstract

**Objective:**

To evaluate the relationship between hospital volume and intermediate‐ and long‐term patient survival for patients undergoing nephrectomy for renal cell carcinoma (RCC).

**Patients and Methods:**

Adult patients with RCC treated with nephrectomy between 2000 and 2010 were identified from the English Hospital Episode Statistics database and National Cancer Data Repository. Patients with nodal or metastatic disease were excluded. Hospitals were categorised into low‐ (LV; <20 cases/year), medium‐ (20–39 cases/year) and high‐volume (HV; ≥40 cases/year), based on annual cases of RCC nephrectomy. Multivariable Cox regression analyses were used to calculate hazard ratios (HRs) for all‐cause mortality by hospital volume, adjusting for patient, tumour and surgical characteristics. We assessed conditional survival over three follow‐up periods: short (30 days to 1 year), intermediate (1–3 years) and long (3–5 years). We additionally explored whether associations between volume and outcomes varied by tumour stage.

**Results:**

A total of 12 912 patients were included. Patients in HV hospitals had a 34% reduction in mortality risks up to 1 year compared to those in LV hospitals (HR 0.66, 95% confidence interval 0.53–0.83; *P* < 0.01). Assuming causality, treatment in HV hospitals was associated with one fewer death in every 71 patients treated. Benefit of nephrectomy centralisation did not change with higher T stage (*P* = 0.17). No significant association between hospital volume and survival was observed beyond the first year.

**Conclusions:**

Nephrectomy for RCC in HV hospitals was associated with improved survival for up to 1 year after treatment. Our results contribute new insights regarding the value of nephrectomy centralisation.

## Introduction

A volume–outcome relationship in surgery was first proposed by Luft in 1979, with the hypothesis that higher surgical volumes and/or greater experience led to lower mortality 1. Since then, numerous studies have reported on the inverse association between hospital volume and patient outcomes in a wide range of surgical and medical procedures. This evidence has led to the implementation of volume‐based referral strategies, most notably by the Leapfrog Group, a watchdog organisation consisting of large corporations and public agencies that purchase healthcare in the USA 2. Similar guidance for urological procedures has also been published in the UK in 2002 in the Improving Outcomes in Urological Cancer Guidance, where centralisation was recommended for radical prostatectomy, cystectomy and in parts for radical nephrectomy where patients have bilateral renal cancer, tumour invading the renal vein or vena cava, or metastatic disease amenable to resection 3. Trends of increasing hospital caseloads have been reported for urological cancer operations in different countries, suggesting the general acceptance of surgical centralisation within the urology community 4, 5, 6. Evidence to support this change for RCC nephrectomy is, however, based on studies that analysed the volume–outcome relationship primarily on short‐term perioperative results, including mortality within 30 days of surgery, complications, length of stay and readmission rates 7. It is currently not well understood how hospital RCC nephrectomy volume is associated with patient survival beyond the immediate postoperative period. Such information can be useful for policy makers and healthcare providers to drive care quality improvements, particularly as the worldwide incidence of RCC is predicted to rise 8. In the present study, we investigate the volume–outcome relationship in renal cancer nephrectomy focusing specifically on survival beyond 30 days.

## Patients and Methods

### Data

Full data were supplied by Public Health England and contain fields from both the English Hospital Episode Statics (HES) database and the National Cancer Data Repository (NCDR). The characteristics of the datasets have previously been described, but in short, these are major administrative databases with full population coverage containing details of all NHS hospital admissions in England, together with tumour‐level records and survival information 4. Ethics approval was granted by the National Research Ethics Committee (reference 15/EM/0340) and Confidentiality Advisory Group (reference 15/CAG/0169).

### Exposure Variables

Patients diagnosed with RCC and treated with either total or partial nephrectomy between 2000 and 2010 were identified using the International Statistical Classification of Diseases and Related Health Problems 10th revision code and Office of Population Censuses and Surveys Classification of Interventions and Procedures version code (Table S1). We excluded patients aged ≤17 years at the time of surgery as well as those treated with nephroureterectomy or nephrectomy of transplanted kidney. A total of 30 763 renal cancer nephrectomies were recorded in the database. We further excluded individuals recorded as having had multiple renal cancer operations or bilateral procedures. In order to control for differences in tumour stage, only patients with complete TNM data were included in our analyses. Patients with nodal or metastatic disease at diagnosis were excluded. We analysed only those surviving beyond the initial 30 days after nephrectomy. All patients were followed up for death until end of 2015.

Patient‐level variables included age, sex, ethnicity and socio‐economic deprivation. Comorbidities at the time of surgery were tabulated using the Royal College of Surgeons Charlson score 9. Type of nephrectomy (radical, partial) and surgical access (open, minimally invasive) were derived from HES procedure codes.

Hospital annual operative caseloads were calculated based on the number of nephrectomies performed by the responsible hospitals. Our previous study found that the median (interquartile range) annual hospital nephrectomy volume in England was 23 (12–39.5) in the year 2010 4. We therefore categorized hospitals into low‐ (LV), medium‐ (MV) and high‐volume (HV) using practical annual thresholds of 20 and 40 cases. All 30 763 RCC nephrectomy cases were used for this calculation.

### Analysis

All statistical analyses were performed using stata 14 10. *P* values <0.05 were taken to indicate statistical significance. Differences in patient characteristics amongst the hospital volume categories were compared using chi‐squared tests or anova. We further explored missing TNM data across hospital volumes and other patient characteristics with chi‐squared tests.

Kaplan–Meier survival estimates of patients treated in different hospital volume categories were generated and compared using log‐rank tests.

Cox proportional hazard regression analyses were used to assess the association between hospital nephrectomy volume and survival. Survival time was calculated from date of surgery until date of death from any cause. Univariable and multivariable models with appropriate adjustment for potential confounders were created. These were identified *a priori* and included age group (≤64 years, 65–74 years, ≥75 years), sex, ethnicity (white, non‐white), socio‐economic deprivation, number of comorbidities, year of nephrectomy (2000–2002, 2003–2005, 2006–2008, 2009–2010), type of nephrectomy, type of surgical access, tumour T stage, tumour grade and histological subtype (clear‐cell, others). Shared frailty Cox models were used to account for clustering of patients within given hospitals and year of nephrectomy. We analysed survival over three follow‐up periods to determine the effects of hospital volume on survival at different post‐surgical intervals. They were defined as short (30 days to 1 year), intermediate (1–3 years) and long (3–5 years) term. Each period was evaluated conditional on patients surviving the previous follow‐up interval. We calculated Harrell's C statistics for each model to assess goodness of fit. When significant improvement is demonstrated for HV hospitals, we quantified the clinical effectiveness by calculating the numbers needed to treat (NNTs) associated with centralisation 11. This number may represent the number of cases that need to be centralised from LV hospitals to HV hospitals in order to prevent one death, assuming that the association is causal.

To examine whether missing TNM data could have affected the estimates of the hospital volume–outcome relationship, we further repeated our analyses only on patients who underwent surgery between 2009 and 2010, where TNM coverage was more complete.

Differences in T stages can have significant impact on the nephrectomy complexity and may therefore benefit differently from surgical centralisation. We therefore performed subgroup analyses based on tumour T stage. To examine whether interaction between hospital volume and T stages contribute significantly to the volume–outcome relationship, we also used the likelihood ratio test to compare multivariable Cox models with and without interaction terms between hospital volume and T stage categories. Due to the small number of patients with T4 disease, these were considered together with T3 patients.

## Results

A total of 12 912 patients were included in the final analyses with a median follow‐up of 8.1 years (Figure S1). A total of 52.7% of eligible patients were excluded because of unrecorded TNM data. Coverage of TNM data varied across hospital volume groups and was less complete for those treated in HV hospitals than for patients treated in LV or MV hospitals, but was increasingly complete in later years of the study up to 69.0% (Table S2).

The mean (range) age of the analysis cohort was 63.4 (19–95) years, and 62.6% were men. The characteristics of the patients treated in each hospital volume group are described in Table 1. There was no difference in the age and sex compositions amongst the hospital volume categories. Patients treated in HV hospitals were more likely to have higher numbers of comorbidities. HV hospitals also performed a higher proportion of partial and minimally invasive surgeries. There were significant differences in the T stage distribution amongst hospital volume categories, with a higher proportion of patients in HV hospitals having T1 disease.

**Table 1:** Patient characteristics stratified by hospital volume category.

	LV hospital (<20 cases)	MV hospital (20–39 cases)	HV hospital (≥40 cases)	*P*
***n***	4468	5309	3135	
**Number of hospitals**	146	96	42	
**Mean (range) age, years**	63.6 (19–93)	63.4 (19–95)	63.2 (20–92)	0.33
**Male, %**	63.2	62.5	61.9	0.51
**White, %**	84.9	88.5	91.7	<0.01
**CCI, %**				
0	64.8	61	58.9	<0.01
1	25.8	27.8	28.1	
≥2	9.4	11.3	13.0	
**IMD, %**				
1 – least deprived	20.3	17.6	20.4	<0.01
2	23.3	22.8	21.9	
3	21.8	23.3	23.0	
4	20.4	20.0	18.5	
5 – most deprived	14.3	16.3	16.1	
**Nephrectomy type, %**				
Radical nephrectomy	95.8	90.1	83.4	<0.01
Partial nephrectomy	4.2	9.9	16.6	
**Minimally invasive access, %**	19.4	33.6	44.8	<0.01
**T Stage, %**				
T1	42.4	47.6	50.9	<0.01
T2	23.6	18.7	15.4	<0.01
T3/T4	34.1	33.8	33.7	0.91
**Grade, %**				
1	11.3	8.6	5.8	<0.01
2	48.6	47.0	45.1	
3	32	34.7	36.4	
4	8.2	9.6	12.7	
**Morphology, %**				
Clear‐cell	92.7	91.0	90.8	0.15
Others	7.3	9.0	9.2	
**Mortality, %**				
1‐year	8.8	7.1	5.4	<0.01
3‐year	20.3	18.3	14.6	<0.01
5‐year	28.6	25.8	22.6	<0.01

CCI, Charlson Comorbidities; HV, high‐volume; IMD, Index of Multiple Deprivation; LV, low‐volume; MV, medium‐volume.

**Figure 1:**
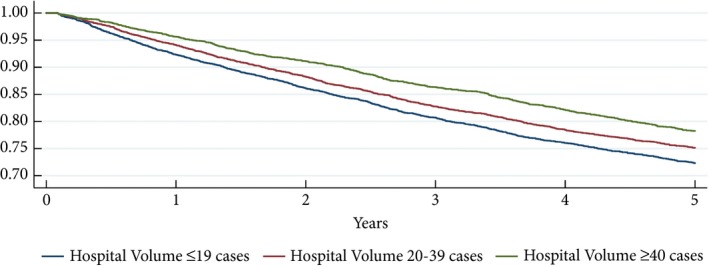
Kaplan–Meier Estimates. Survival estimates for patients with RCC treated with nephrectomy by hospitals with different annual nephrectomy volumes.

Patients in HV hospitals had significantly lower crude unadjusted mortality rates at 1, 3 and 5 years. Kaplan–Meier analysis also showed improved survival of between 30 days and 5 years after nephrectomy for patients treated in HV hospitals (*P* < 0.01; Fig. 1).

### Short‐Term Survival (3 days to 1 year)

**Figure 2:**
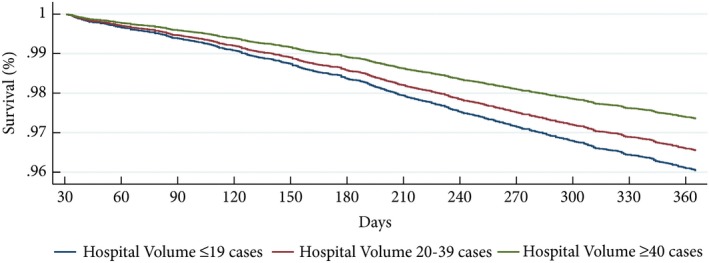
Predicted patient survival probability between 3 days and 1 year post RCC nephrectomy for patients treated by hospitals with different annual volumes.

In the univariable model, treatment in HV centres resulted in significantly better outcomes compared to treatment in LV centres (hazard ratio [HR] 0.57, CI 0.46–0.70; *P* < 0.01 [Table 2]). After adjusting for covariates, patients treated in HV centres continued to have better outcomes, with a 34% reduction in mortality hazard (HR 0.66, CI 0.53–0.83; *P* < 0.01 [Table 2]). A typical patient in HV hospitals had a predicted survival probability of 97.4% at 1 year, compared to 96.0% for a patient in LV hospitals, corresponding to a NNT of 71 (Fig. 2).

**Table 2:** Univariable and multivariable Cox proportional hazard regression models with shared frailty examining conditional survival over three follow‐up periods for patients treated in three hospital nephrectomy volume categories. Restricted cohort consisted of patients treated in 2009 and 2010 and analysed separately, showing consistent results.

Variables	Short (3 days to 1 year)			Intermediate (1–3 years)			Long (3–5 years)		
	HR	95% CI	*P value*	HR	95% CI	*P* value	HR	95% CI	*P value*
**Univariable model**									
** Hospital volumes**									
<20 cases	Reference			Reference			Reference		
20–39 cases	0.77	0.66–0.90	<0.01	0.96	0.84–1.09	0.5	0.88	0.76–1.02	0.09
≥40 cases	0.57	0.46–0.70	<0.01	0.75	0.64–0.88	<0.01	0.89	0.75–1.06	0.2
C–Index	0.56 (0.53–0.58) *P* < 0.01			0.53 (0.51–0.54) *P* < 0.01	0.51 (0.50–0.53) P < 0.01		
**Multivariable model**									
**Hospital volumes**									
<20 cases	Reference			Reference			Reference		
20–39 cases	0.87	0.74–1.03	0.11	1.05	0.93–1.20	0.42	0.95	0.81–1.11	0.50
≥40 cases	0.66	0.53–0.83	<0.01	0.86	0.73–1.01	0.07	1.01	0.83–1.22	0.93
**Age**									
64 years	Reference			Reference			Reference		
65–74 years	1.19	1.00–1.41	0.04	1.31	1.16–1.49	<0.01	1.41	1.21–1.64	<0.01
≥75 years	1.51	1.25–1.81	<0.01	1.76	1.54–2.01	<0.01	2.43	2.08–2.84	<0.01
**Sex**									
Male	Reference			Reference			Reference		
Female	1.09	0.93–1.27	0.28	0.93	0.83–1.04	0.22	0.84	0.73–0.96	0.01
**Ethnicity**									
White	Reference			Reference			Reference		
Non–white	0.80	0.54–1.16	0.24	0.85	0.65–1.11	0.24	0.74	0.54–1.02	0.07
**CCI**									
0	Reference			Reference			Reference		
1	2.00	1.69–2.37	<0.01	1.75	1.55–1.97	<0.01	1.39	1.20–1.60	<0.01
≥2	3.39	2.79–4.13	<0.01	2.35	2.02–2.73	<0.01	2.13	1.78–2.55	<0.01
**IMD**									
1	Reference			Reference			Reference		
2	1.06	0.84–1.33	0.63	0.94	0.80–1.11	0.48	1.02	0.84–1.25	0.81
3	1.11	0.88–1.39	0.37	1.03	0.87–1.21	0.72	1.03	0.84–1.25	0.80
4	1.28	1.02–1.60	0.04	1.05	0.89–1.24	0.57	1.18	0.96–1.45	0.11
5	1.06	0.81–1.37	0.67	1.04	0.87–1.25	0.64	1.26	1.02–1.56	0.03
**Year**									
2000–2002	Reference			Reference			Reference		
2003–2005	0.82	0.65–1.04	0.11	0.88	0.73–1.11	0.16	0.78	0.62–0.99	0.04
2006–2008	0.79	0.62–1.00	0.05	0.74	0.61–0.89	<0.01	0.78	0.62–0.98	0.03
2009–2010	0.67	0.52–0.88	<0.01	0.65	0.53–0.79	<0.01	0.70	0.55–0.89	<0.01
**T stage**									
1	Reference			Reference			Reference		
2	1.54	1.19–2.00	<0.01	1.62	1.37–1.91	<0.01	1.34	1.12–1.61	<0.01
3	3.13	2.54–3.85	<0.01	2.61	2.27–3.00	<0.01	1.92	1.65–2.23	<0.01
4	11.65	8.30–16.37	<0.01	4.00	2.64–6.06	<0.01	3.56	2.06–6.15	<0.01
**Grade**									
1	Reference			Reference			Reference		
2	0.66	0.45–0.95	0.03	1.20	0.91–1.58	0.21	0.94	0.72–1.22	0.62
3	1.45	1.01–2.08	0.04	1.74	1.32–2.31	<0.01	1.34	1.03–1.75	0.03
4	2.50	1.71–3.65	<0.01	3.45	2.56–4.64	<0.01	1.49	1.08–2.05	0.02
**Morphology**									
Clear‐cell	Reference			Reference			Reference		
Others	1.00	0.59–1.71	0.99	1.19	0.86–1.65	0.29	1.10	0.75–1.61	0.62
**Nephrectomy type**									
Radical	Reference			Reference			Reference		
Partial	0.33	0.18–0.59	<0.01	0.58	0.43–0.77	<0.01	0.52	0.38–0.71	<0.01
**Surgical access**									
Open	Reference			Reference			Reference		
Minimally invasive	0.69	0.56–0.85	<0.01	0.71	0.61–0.81	<0.01	0.77	0.66–0.90	<0.01
C–Index	0.79 (0.78–0.80) *P* < 0.01			0.73 (0.72–0.75) *P* < 0.01			0.70 (0.69–0.72) *P* < 0.01		
**Multivariable model (2009–2010 Cohort)**									
**Hospital volumes**									
<20 cases	Reference			Reference			Reference		
20–39 cases	0.72	0.50–1.03	0.07	1.13	0.84–1.53	0.42	1.18	0.84–1.66	0.33
≥40 cases	0.64	0.43–0.94	0.02	0.95	0.69–1.31	0.75	1.22	0.86–1.75	0.27
C–Index	0.80 (0.77–0.83) *P* < 0.01			0.75 (0.73–0.77) *P* < 0.01			0.74 (0.72–0.77) *P* < 0.01		

CCI, Charlson Comorbidities; IMD, Index of Multiple Deprivation.

As expected, being older, having a higher number of comorbidities, higher tumour T stage and grade were associated with poorer outcomes. Treatment with partial nephrectomy or minimally invasive surgery and treatment in later periods were predictors of improved survival.

### Intermediate (1–3 years) and Long‐term (3–5 years) Survival

Beyond the first postoperative year, there was no evidence that surviving patients treated in MV or HV hospitals had different outcomes compared to LV hospitals (Table 2). Older age, greater number of comorbidities, and higher tumour T stage and grade remained significant predictors of lower survival, while operations in more recent years and with nephron‐sparing surgery and minimally invasive access continue to be predictors of the reverse. Social deprivation did not consistently affect patient survivals.

### Restricted Cohort

To examine whether missing TNM data affected our results, we repeated our analyses on patients treated between 2009 and 2010. In this restricted cohort, TNM data were available for 69.0% of the eligible patients, compared to 47.3% in the entire cohort.

Results remained consistent with patients treated in HV hospitals having a 36% reduction in mortality hazard in the first year compared with those treated in LV hospitals (HR 0.64, CI 0.43–0.94; *P* = 0.02 [Table 2]). No association between hospital volume and survival was observed after 1 year.

### T Stage Subgroup Analyses

During the short‐term follow‐up period between 3 days and 1 year, treatment in HV hospitals was found to improve survival for patients with T1 (HR 0.57, CI 0.33–0.97; *P* = 0.04) and T2 disease (HR 0.41, CI 0.20–0.83; *P* = 0.01 [Table 3]). These corresponded to NNTs of 143 and 37, respectively, assuming causality. No significant association was observed between hospital volume and survival for patients with T3 or T4 disease or beyond the first year of follow‐up. However, the difference between ‘significant’ and ‘not significant’  is not itself statistically significant and when examined using likelihood ratio test, there was no statistical evidence of interaction between hospital volume and T stage categories (*P* = 0.17).

**Table 3:** Multivariable Cox proportional hazard regression models with shared frailty examining conditional survival over three follow up periods for patients with localised RCC stratified by T stages

	Hospital volume	Short (3 days to 1 year)			Intermediate (1–3 years)			Long (3–5 years)		
		HR	95% CI	*P*	HR	95% CI	*P*	HR	95% CI	*P*
T1	<20	Reference			Reference			Reference		
	20–39	0.69	0.45–1.04	0.07	1.03	0.79–1.36	0.82	1.04	0.81–1.35	0.76
	≥40	0.57	0.33–0.97	0.04	0.88	0.62–1.26	0.49	0.98	0.70–1.37	0.89
T2	<20	Reference			Reference			Reference		
	20–39	0.55	0.36–0.84	0.01	0.96	0.73–1.26	0.76	0.84	0.60–1.17	0.30
	≥40	0.41	0.20–0.83	0.01	0.65	0.44–0.95	0.03	0.93	0.60–1.46	0.76
T3/T4	<20	Reference			Reference			Reference		
	20–39	0.98	0.79–1.21	0.87	1.09	0.92–1.29	0.31	0.91	0.71–1.15	0.42
	≥40	0.77	0.58–1.02	0.07	0.90	0.73–1.11	0.31	1.05	0.79–1.39	0.75

HR, hazard ratio.

## Discussion

In the present study, we found that a survival benefit exists for RCC patients treated in HV hospitals up to 1 year after nephrectomy. Benefit beyond the first year of follow‐up was not observed. Patient age, number of comorbidities, tumour T stage and grade at time of surgery remained significant predictors of long‐term survival, although there was no evidence that particular T stage benefitted preferentially from surgical centralisation.

The effect of hospital volume on RCC patient outcomes has been a subject of interest and well characterised in widespread publications in recent years 12, 13, 14, 15. Most of these studies have concluded that higher hospital volume correlates to better outcomes, particularly during the perioperative period. Our previous meta‐analysis on the effect of radical nephrectomy centralisation showed that HV hospitals reduced surgical mortality risks by 26% and complications by 18% compared to LV hospitals 7. Because of the relatively low mortality rates associated with RCC nephrectomy, we suggested that the perceived clinical benefit of centralisation was however marginal. Longer‐term survival in this instance may therefore represent a more appropriate outcome measure, particularly as 1‐ and 5‐year survival are more often used as benchmarks for assessing the efficacy of oncological treatment.

Similar findings of improved long‐term oncological outcomes in HV centres have been reported in other cancer resections, including those for breast and oesophageal cancer and pancreatic ductal adenocarcinoma 16, 17, 18. Hospital volume should however be viewed as a proxy measure for healthcare quality, encompassing the quality of surgical treatment and pre‐ and postoperative care, but does not in itself identify the exact aspects and practices of HV hospitals that drive the observed association with survival. There are several mechanisms that could explain our findings. First, surgeons in HV hospitals have greater experience and skills in RCC nephrectomy resulting in lower periperative morbidity and therefore reduced 1‐year mortality. Second, HV hospitals may also have more streamlined periperative pathways that contribute to the improved short‐term outcomes. Third, greater patient exposure by the complementary multidisciplinary team including radiologists and oncologists may also lead to improvements in detecting disease recurrence facilitating more timely interventions. Fourth, larger centres also often have the required infrastructure to enable quicker adoption of new clinical guidelines and greater access to novel treatments including clinical trials, although this may not be as relevant in the present cohort, which focused only on patients with localised disease at time of nephrectomy. The association between hospital volume and survival attenuated after adjustments for patient‐level characteristics, suggesting a role of case‐mix in explaining the crude variation, but also indicating that case‐mix alone is unlikely to explain the whole difference.

Our findings are suggestive of the presence of a volume–outcome relationship for RCC nephrectomy across volume categories, with patients treated by hospitals with higher activity having lower risks of mortality, consistent with previous studies examining volume–outcome relationships in patients with lung, bladder and stomach cancer 19, 20. The body of previous evidence and our own findings support the hypothesis that centralising nephrectomy activity for RCC is associated with improved survival beyond the immediate postoperative period.

To further contextualize the effect of surgical centralisation on RCC nephrectomy, we calculated the NNT. Assuming causality and that patients treated in LV centres had the same outcomes as those treated in HV centres, prevention of one death in the first year could be realised after centralising 71 patients. This is substantially lower than the 234 patients estimated in our previous review that would be required to be centralised to prevent one periperative death, and may therefore represent stronger evidence to support RCC nephrectomy centralisation 7.

A positive volume–outcome relationship is more likely to be observed in technically challenging procedures that carry higher morbidities and mortalities. It is therefore surprising that T stage did not affect the benefit observed with nephrectomy centralisation in our analyses, particularly as T3 and T4 stages represent advanced disease with perinephric invasion. It is plausible that patients with T3 and T4 disease have generally poorer prognosis, diminishing the effect of hospital volumes, as evident in the higher point estimate for the HR. Our stage‐specific sample size may also not be sufficient to detect statistical significance in individual subgroups.

To the best of our knowledge, this is currently the only study to describe the association between hospital volume and RCC nephrectomy survival beyond the immediate postoperative period. Other strengths of the present study include the use of data with whole population coverage, with all patients followed up for a minimum of 5 years. Patient reporting in the HES and NCDR databases is mandatory, leading to case ascertainment of >98% 21. Linkage of the two databases also allowed patient‐, hospital‐ and tumour‐level variables to be included in our statistics model, resulting in improved model fits. Using patient data from England where the healthcare system relies more on regional network referral and where patients have less choice about their treatment doctors, selective referral whereby high‐performing hospitals are more likely to receive further cases, is less likely to be a confounding factor.

The present study has several limitations. There is potential selection bias as a significant proportion of the cohort had incomplete TNM records; however, we observed consistent results when analyses were repeated on a restricted cohort with significantly more complete TNM data coverage. Use of adjuvant or neoadjuvant systemic therapy was not recorded in the NCDR and was therefore not adjusted in our analyses. While systemic therapy can have a significant impact on long‐term survival, this has not traditionally been advocated for patients with localised disease, on which our study focused exclusively. We measured the effect of hospital volume on overall survival, but did not control for differences in background mortality. It is possible that patients treated by different volume hospitals had different non‐RCC mortality risks, although adjustment for comorbidities mitigated these concerns.

Future research should focus on identifying the exact quality and process of care in HV centres that drive outcome improvements, allowing lower‐performing hospitals to adopt these practices. In the era of centralisation, guidelines have been published on the volume threshold that hospitals should achieve annually. Yet, these have been based on very limited evidence and there is currently no consensus on the definition of an HV hospital. More study should therefore focus on ascertaining the minimum number of nephrectomies that providers should carry out to attain acceptable outcomes.

In the present analysis of volume–outcome relationship, we further characterised the survival benefit of RCC nephrectomy centralisation and found that improvement exists beyond the immediate perioperative period and extends to 1 year after surgery. Our results contribute to the growing evidence in support of nephrectomy centralisation for patients with RCC.

## Conflict of Interest

None declared.

## Supporting information


**Figure S1**. Flow diagram for patient selection.

**Table S1**. ICD10 and OPCS4 codes used to identify patient cohort.

**Table S2**. Completeness of TNM stage data by patient characteristics.
