# Supplementary material for: Impact of hospital nephrectomy volume on intermediate‐ to long‐term survival in renal cell carcinoma
Source: BJU Int. 2019 Jul 15;125(1):56–63. doi: 10.1111/bju.14848 (PMC6973244; doi:10.1111/bju.14848)
Supplement: Supplementary file 1 — Figure S1. Flow diagram for patient selection. [file BJU-125-56-s001.docx]

Supplementary Figure 1: Flow diagram for patient selection.

1,164 patients with nodal or metastatic disease excluded.

15,671 records excluded due to missing TNM stage.

1,016 records excluded

933 multiple nephrectomies

67 bilateral nephrectomies

16 multiple nephrectomy types

Number of patients included in final analyses. n=12,912

Number of patients with complete TNM stage. n=14,076

Number of patients with single nephrectomy. n=29,747

Number of nephrectomy records. n=30,763
