# Supplementary material for: Impact of hospital nephrectomy volume on intermediate‐ to long‐term survival in renal cell carcinoma
Source: BJU Int. 2019 Jul 15;125(1):56–63. doi: 10.1111/bju.14848 (PMC6973244; doi:10.1111/bju.14848)
Supplement: Supplementary file 2 — Table S1. ICD10 and OPCS4 codes used to identify patient cohort. [file BJU-125-56-s002.docx]

**Supplementary Table 1.** ICD10 and OPCS4 codes used to identify patient cohort

**ICD10 Codes**

Renal Cell Carcinoma – C64

**OPCS4 Codes**

Total excision of kidney – M021, M023, M024, M025, M028, M029

Partial excision of kidney – M031, M032, M038, M039

Nephroureterectomy (excluded) – M022

Excision of transplanted kidney (excluded) – M026, M027

Laparoscopic/Robotic Access – Y508, Y751, Y752, Y753, Y754, Y755, Y758, Y759
